# Reply to Formaldehyde Emissions From 2‐Octyl Cyanoacrylate: Quantified Risk for Allergic Contact Dermatitis

**DOI:** 10.1111/cod.70235

**Published:** 2026-09-08

**Authors:** Mehrdad Mark Mofid

**Affiliations:** ^1^ Department of Plastic and Reconstructive Surgery Johns Hopkins University School of Medicine Baltimore Maryland USA

To the Editor,

We thank Tomaszewski and colleagues for their careful reading of our study [1]. To clarify, our two independently prepared airborne replicates agree to within 10%, demonstrating reproducibility rather than population variability. The chamber values describe two‐hour device emissions, so comparison with the National Institute for Occupational Safety and Health (NIOSH) eight‐hour limit is extrapolated. Humidity and equilibration time went unreported, though temperature and air exchange did not. Our scenarios assume emission does not decay, making the constant‐flux case an upper bound. The Environmental Protection Agency (EPA) 1 μg/cm^2^ value is a precautionary screening benchmark, not a device acceptance criterion, and our study was never framed as an International Organization for Standardization (ISO) 10993 biological evaluation.

Three objections by Tomaszewski et al. are invalid. The first concerns benchmarks. The letter treats EPA and human elicitation data as a single comparator. The EPA figure is a precautionary benchmark, whereas Flyvholm et al. [2] and Fischer et al. [3] report observed elicitation doses from controlled dose–response studies in patch‐test‐confirmed formaldehyde‐allergic patients. Both are peer‐reviewed human dose–response studies, and Fischer et al. standardized the TRUE Test formaldehyde patch still used clinically. They are the appropriate comparator here.

The second concerns ventilation. The letter's comparators are the operating room, where ANSI/ASHRAE/ASHE Standard 170 [4] requires at least 20 air changes per hour, and the recovery area. Dermabond Prineo remains on the skin for 7–14 days per its instructions for use, of which only a few hours fall in the operative and recovery period. The remainder is spent in domestic settings, where measured residential air exchange has a median near 0.7 air changes per hour, representative of our testing [5].

The third concerns hydrolysis. The letter treats the formaldehyde we measured as an artefact of the humidified chamber, assuming skin would not supply comparable moisture. Water both initiates polymerization and liberates formaldehyde from the cured polymer, a process Park et al. [6] measured in vitro. A fresh incision beneath an occlusive film is supplied continuously with serous exudate, transepidermal water loss, and perspiration, so our chamber likely understated both the available water and the formaldehyde released.

Moving to clinical data, Richter et al. [7] used a split‐incision design to compare the mesh system and intradermal sutures in Prineo's initial trial. Equivalence was declared on wound approximation. There was no hypersensitivity endpoint, and the inflammatory response instrument scored erythema, edema, pain and temperature, with no vesicular component. Blistering was tabulated separately and only for the device arm: none at 24 h, two of 83 patients at Day 7, and two of 83 at days 12 to 25. Injury from application would present immediately, so a later presentation favours delayed hypersensitivity. However, no patch testing was reported, so allergic contact dermatitis (ACD) cannot be confirmed.

More recent studies report a consistent signal. Koritz et al. [8] found skin reactions in 12% of 234 paediatric orthopaedic procedures after a single application, rising to 21% on re‐exposure; Jones et al. [9] found reactions in 1.9% of arthroplasty patients without prior exposure and 8.1% of those with; Ali and Makhdom [10] found 1% after unilateral total knee arthroplasty and 22% after the second side in staged bilateral cases. Dose–response on re‐exposure is the signature of sensitization. Where patch testing was performed it confirmed the diagnosis of ACD: Ricciardo et al. [11] reported positive reactions to Dermabond Prineo glue in all six patients tested, five with prior Dermabond exposure. The same was true outside of orthopaedics. In a prospective series of 102 consecutive breast surgery patients, Nigro et al. [12] observed dermatitis in 14%, with every reaction confirmed by scratch testing to the adhesive. Beyond this, reactions after a first surgical exposure are also well described [13], consistent with community exposure to nail and eyelash adhesives.

The post‐market record points the same way. FDA MAUDE holds 4622 unique report keys naming a Dermabond‐brand 2‐octyl cyanoacrylate device. Of these, 2127 (46%) name Dermabond Prineo, a share that reaches 56% for reports received since 2015. Annual Prineo counts roughly tripled, from 92 in 2015 to 282 in 2025. Among the Prineo reports the coded content is overwhelmingly cutaneous, led by skin inflammation or irritation, rash, hypersensitivity, erythema, pruritus and blistering, while device failure codes are nearly absent. Adverse events of this kind reach the FDA through the manufacturer, so the pattern described here was available to Ethicon. Patient‐reported data show the same pattern. In a structured review of a single social media platform within the public domain, Reddit posts from 2009 to 2026 we identified 1992 unique self‐reported adverse events attributed to 2‐octyl cyanoacrylate skin closure, of which 1357 (68%) described features of ACD, 537 reported seeking clinical care and 140 met a serious‐outcome definition. Among the 616 posts stating a time to onset the median was 7 days, matching the interval at which blistering appeared in Richter's device arm and again pointing to delayed hypersensitivity rather than immediate irritation. In agreement with these findings, a prior systematic review and MAUDE analysis reported the same predominance of cutaneous hypersensitivity events [14].

To conclude, we agree that bench data do not predict clinical risk. On that standard, the clinical evidence is substantial and consistent, if not overwhelming, and that risk is plausibly attributable to chemical exposure at the skin surface, including but not limited to formaldehyde. Quantifying that exposure was the purpose of our study. To our knowledge, this remains the only such measurement in the literature; no comparable data on formaldehyde emissions from 2‐octyl cyanoacrylate products have been published by the manufacturer. These findings identify a potential exposure that is not described in current product labelling and that may be relevant to clinicians evaluating patients with suspected reactions to this adhesive.

## Author Contributions

**Mehrdad Mark Mofid:** conceptualization, writing – review and editing, writing – original draft.

## Funding

The author has nothing to report.

## Conflicts of Interest

Mehrdad Mark Mofid, MD is an officer and a shareholder of Sylke Inc.

## Data Availability

The data that support the findings of this study are available from the corresponding author upon reasonable request.
